# A rare case of hydatid cyst presenting as a breast cancer. A case report^☆^

**DOI:** 10.1016/j.ijscr.2024.110787

**Published:** 2024-12-28

**Authors:** Misganaw Ayenew Amogne, Muluken Getinet Mekuriaw, Dagimawi Abiy Abate, Yabibal Tsegaye Workineh, Mandante Bogale Tiruneh

**Affiliations:** a Debre Markos University, Pathology Department, Ethiopia; b Debre Markos University, Pediatrics and Child Health Department, Ethiopia; c Debre Markos University, Surgery Department, Ethiopia

**Keywords:** Hydatid cyst, Cytological findings,, Benign breast lesion,, Parasitic infection, Diagnostic challenges, Case report

## Abstract

**Introduction and importance:**

Hydatid disease, caused by the Echinococcus parasite, is a significant health concern in endemic regions. While commonly found in the liver and lungs, breast involvement is rare. We present a case of a hydatid cyst in the breast of a 34-year-old woman from Ethiopia, initially suspected to be breast cancer.

**Case presentation:**

A 34-year-old woman was admitted to our Hospital with a progressive, painless swelling in her left breast that had persisted for nine months. The patient first noticed the mass while showering and reported no associated symptoms such as discharge, skin changes, or ulceration. There was no family history of breast cancer, trauma, or breast surgery. Physical examination revealed a non-tender, hard mass measuring 6 by 8 cm in the upper outer quadrant of the left breast, fixed to the skin without overlying skin changes.

**Clinical discussion:**

The initial clinical suspicion was breast cancer. Further evaluation included FNAC, which yielded crystal-clear fluid containing laminated metachromatic materials. Ultrasound imaging revealed an anechoic cystic mass with a double-layered wall and posterior acoustic enhancement. Surgical intervention was performed to obtain a definitive diagnosis. Intraoperatively, a 4 by 6 cm cystic mass was identified, adherent to the surrounding breast tissue. The final diagnosis was confirmed as a hydatid cyst of the breast.

**Conclusion:**

This case underscores the importance of considering differential diagnoses in patients presenting with breast masses, particularly in endemic regions for hydatid disease. Awareness of such atypical presentations can lead to timely and appropriate management.

## Highlights

• Hydatid disease presenting as a breast mass in a 34-year-old woman, initially suspected to be breast cancer.
• Atypical presentations of hydatid cysts, emphasizing the need for differential diagnosis in endemic regions.
• fine-needle aspiration cytology and ultrasound imaging will facilitate accurate diagnosis of unusual hydatic cyst.
• surgical excision, leads to a definitive diagnosis and successful management of hydatid cyst.
• It is very important to consider hydatid disease in the differential diagnosis of breast masses, in endemic areas.

## 1 Introduction

Hydatid disease, caused by the larval stage of the Echinococcus parasite, is a significant public health concern in many parts of the world, particularly in regions where livestock farming is prevalent [1,2]. The most common species involved in human infections are Echinococcus granulosus and Echinococcus multilocularis. While hydatid cysts predominantly affect the liver and lungs, they can also occur in unusual sites, including the breast, leading to diagnostic challenges. The incidence of hydatid cysts in the breast is 0.27 % of all reported cases. [[3], [4], [5]].

Breast masses are a common clinical finding, with a wide differential diagnosis that includes benign conditions such as cysts and fibroadenomas, as well as malignant tumors [3,6]. The presentation of a hydatid cyst in the breast is rare, and its symptoms may mimic those of more common breast pathologies. Patients may present with a palpable mass, localized pain, or changes in breast contour. Imaging studies, including ultrasound and mammography, may reveal cystic lesions but often do not provide definitive diagnosis [[7], [8], [9]].

The management of hydatid cysts typically involves surgical intervention, particularly in cases where the cysts are symptomatic or cause complications. However, due to the rarity of breast hydatid cysts, there is limited literature addressing their specific clinical management and outcomes [3,10].

Understanding the presentation and management of hydatid cysts in the breast is crucial for clinicians to avoid misdiagnosis and ensure appropriate treatment [10]. This case report aims to highlight a rare instance of a hydatid cyst presenting as a breast mass, discussing its clinical features, diagnostic approach, and therapeutic considerations.

## 2 Case presentation

This is a 34 year old para II women presented with a progressive painless left breast swelling of 9 month duration which she noticed while showering but no history of discharge overlying skin change or ulceration, she has no family history of breast cancer, she has no history of trauma or breast surgery. For this presentation she was admitted to Debremarkos comprehensive specialized hospital on Feb 2024.

On physical examination there is 6 by 8 cm non tender hard mass fixed to the skin but no overlying skin change or discharge on the upper outer quadrant. For this presentation she was admitted to the hospital for presumptive diagnosis of breast ca.

On laboratory examination FNAC shows a crystal-clear fluidal aspirate composed of a few laminated metachromatic materials on ultrasound an anechoic cystic mass having a double-layered wall with posterior acoustic enhancement was appreciated for the diagnosis of breast cyst operation was done and intraoperatively 4 by 6 cm cystic mass with is adherent to the breast tissue was found. This case first was approached as a breast cancer but latter it becomes a hydatic cyst lesion of breast.

## 3 Investigations

1. FNAC

Crystal-clear fluidal aspirate composed of a few laminated metachromatic materials, there are no cells

2. Breast Ultrasound:

An anechoic cystic mass having a double-layered wall with posterior acoustic enhancement was appreciated.

3. Histopathologic findings

A fragment of a hydatid cyst with eosinophilic laminated layers, chronically inflamed pericyst infiltrated with eosinophil-dominant, mixed chronic inflammatory exudates and multiple protoscolices.

N.B A blood test for echinococcosis, CT and MRI were not available in our hospital and were not conducted for this patient.

## 4 Discussion

This case highlights the importance of thorough clinical evaluation and differential diagnosis in patients presenting with breast masses. The initial presentation of a progressive, painless left breast swelling in a 34-year-old woman raised significant concern for breast cancer, given the characteristics of the mass and the absence of benign symptoms such as discharge or skin changes. This concern was further supported by the mass's size (6 × 8 cm), firmness, and fixation to underlying tissues, which are often red flags in breast examinations.

However, the subsequent diagnostic workup revealed a surprising etiology: a hydatid cyst of the breast. Hydatid disease, caused by the Echinococcus species, is typically associated with cyst formation in organs such as the liver and lungs, but it can also manifest in unusual locations, including the breast. This case underscores a critical point in medical practice: while malignancy is often the primary concern in breast masses, rare conditions such as hydatid cysts must also be considered, especially in regions where echinococcosis is endemic.

The FNAC results showing crystal-clear fluid and laminated metachromatic materials were pivotal in guiding the diagnosis away from malignancy. While FNAC is an excellent tool for evaluating breast masses, its limitations must be acknowledged [3,5]. In this case, the cytological findings were atypical for breast cancer and suggested a non-malignant process. The ultrasound findings of an anechoic cystic mass with a double-layered wall and posterior acoustic enhancement further indicated a cystic lesion rather than a solid tumor [9].

Imaging techniques such as CT and MRI play a vital role in diagnosing hydatid cysts and differentiating them from other cystic lesions. CT and MRI provide high resolution images that allow for the visualization of cysts with greater sensitivity and specificity compared to other imaging modalities like ultrasound. Both CT and MRI can help characterize the cysts based on their appearance, size, location, and the presence of complications such as rupture or secondary infection. These imaging techniques can identify complications associated with hydatid disease, such as cyst rupture, anaphylactic reactions, or secondary infections. Accurate imaging helps in surgical planning by providing detailed anatomical information about the cysts and surrounding structures. CT and MRI were not available in our hospital and were not conducted for this patient [3,5,9].

The management of hydatid cysts, primarily caused by Echinococcus species, depends on factors such as cyst location, size, symptoms, and the patient's overall health. Options include:

1. Observation: For asymptomatic patients with small, uncomplicated cysts, regular imaging follow-up may suffice.
2. Medical Management: Antiparasitic medications like albendazole can reduce cyst size and are particularly useful for multiple cysts or high-risk surgical patients. This can be administered pre or on postoperative period.
3. Surgical Management: Often necessary for symptomatic or complicated cysts. Options include:
  • Cystectomy: Removal of the cyst and its membrane.
  • Pericystectomy: Removal of the cyst with surrounding tissue to reduce recurrence.
  • Laparoscopic Surgery: Minimally invasive approach for select cases.
  • Drainage Procedures: For infected or ruptured cysts, percutaneous aspiration with sclerotherapy may be used.
  • Open Surgery: Required for larger or complicated cysts.

In our case, due to limited resources and trained professionals, we opted for an open surgical approach. Intraoperatively, we confirmed a cystic mass adherent to breast tissue, leading to the diagnosis of a hydatid cyst. Surgical excision was performed, highlighting the need for clinicians to consider a broad differential diagnosis when evaluating breast masses, especially in patients without significant risk factors for breast cancer [2,9,10].

This case illustrates the importance of considering geographic and environmental factors in the diagnosis of unusual conditions. Hydatid disease is more prevalent in certain regions, and awareness of its potential manifestations can aid in timely and accurate diagnosis.

## 5 Conclusion

In conclusion, this case serves as a reminder of the complexities involved in diagnosing breast masses. It emphasizes the necessity for comprehensive evaluation and consideration of both common and rare conditions. Clinicians should remain vigilant and open-minded when interpreting clinical findings, as this may ultimately lead to better patient outcomes. The multidisciplinary approach involving radiologists, pathologists, and surgeons is essential in managing such cases effectively.

### 5.1 Learning point

This case underscores the importance of including hydatid cysts in the differential diagnosis of breast masses, both benign and malignant. Recognizing hydatid cysts early can prevent potential complications associated with diagnostic procedures such as fine-needle aspiration cytology (FNAC) and surgical interventions. If the presence of a hydatid cyst is not considered, there is a risk of anaphylactic shock due to the leakage of cystic fluid during these procedures, which can contain allergens and antigens from the Echinococcus parasite.

By maintaining a high index of suspicion for hydatid disease in patients presenting with breast masses, clinicians can avoid life-threatening complications and ensure timely and appropriate management. Early identification and intervention are crucial to prevent leakage of cyst contents, which could lead to severe systemic reactions and further complications.

In summary, awareness of hydatid cysts as a potential cause of breast masses is essential for safe clinical practice and effective patient care (Figs. 1, 2, 3, 4, 5, 6, 7, 8, 9, 10).

**Figs. 1 and 2:**
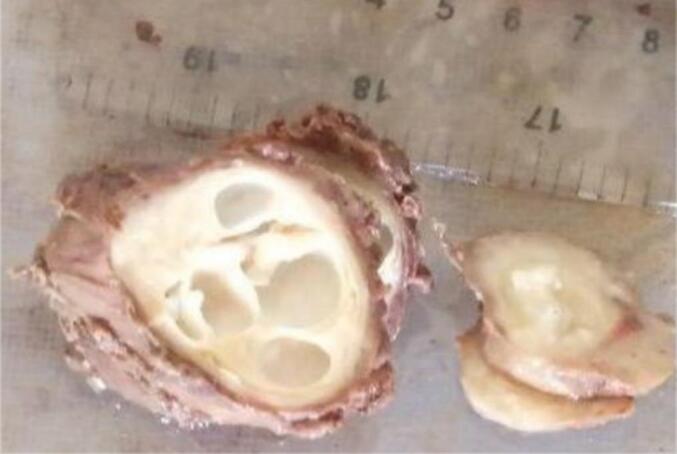

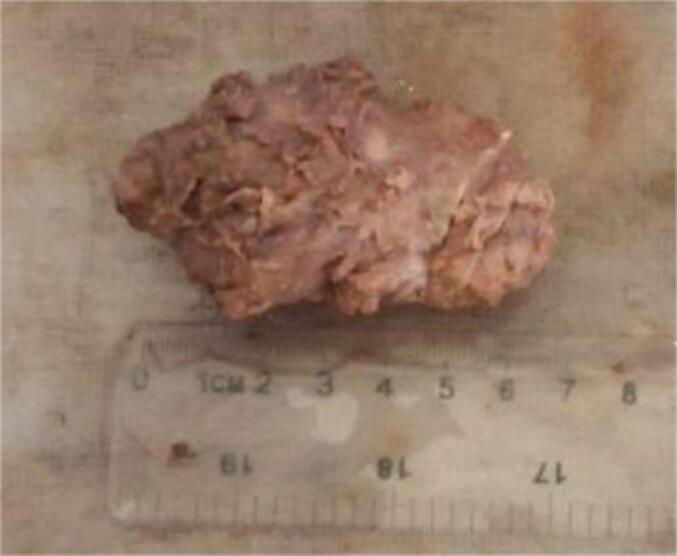
Specimen dimensions; the specimen measures approximately 4 cm by 6 cm. The mass is encapsulated and has a smooth, glistening surface. It exhibits a multilobulated morphology, indicating the presence of multiple cystic structures. The external surface is translucent. Upon sectioning, the cut surface reveals multiple cystic cavities filled with clear, serous fluid. The cyst walls are thin and semi-translucent, consistent with hydatid cysts.

**Figs. 3–6:**
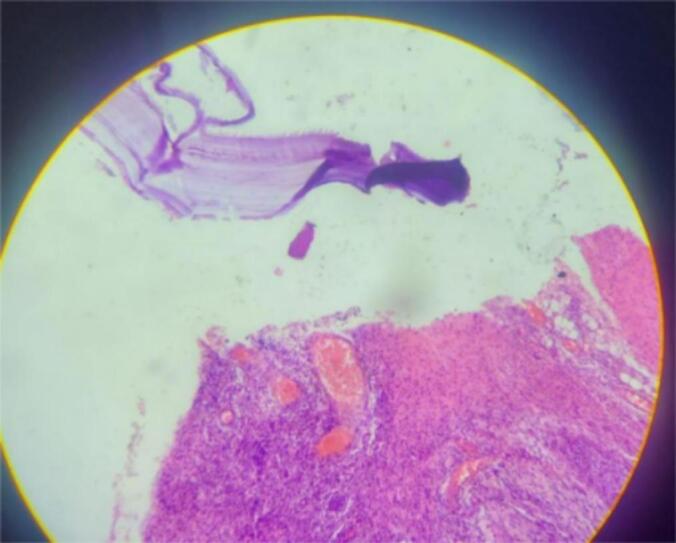

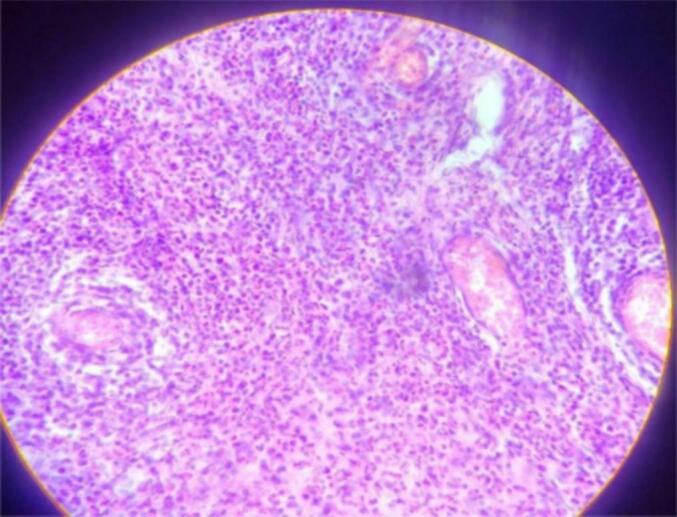

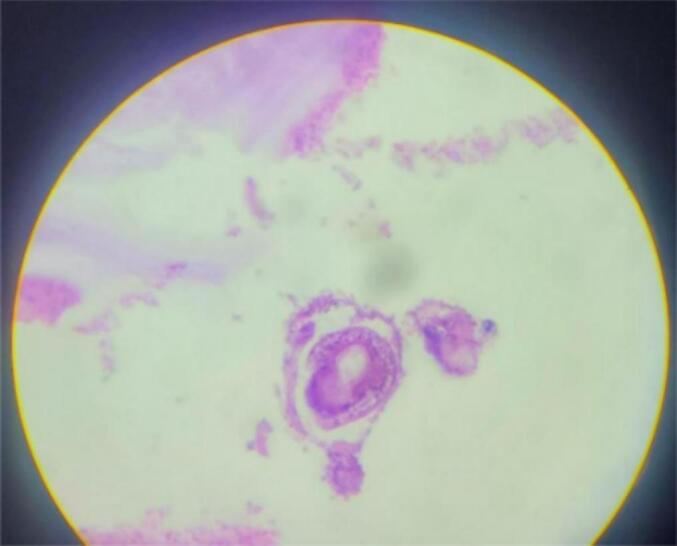

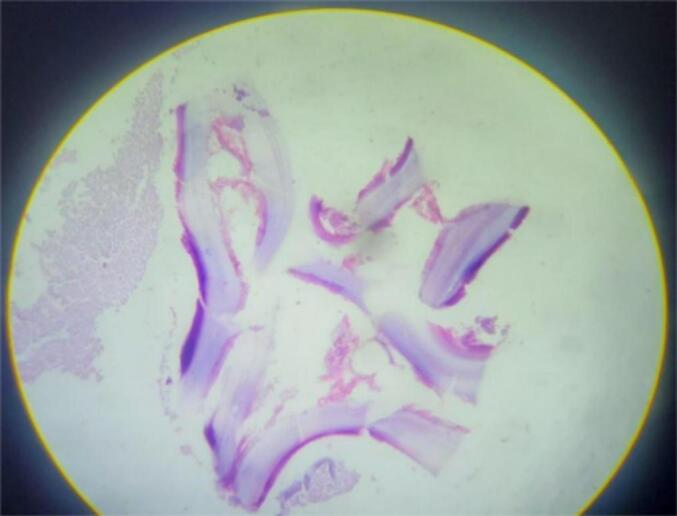
Are a histopathological examination slide pictures. The description will be as follows. Fig. 3 and 6 - a fragment of a hydatid cyst with eosinophilic laminated layers. Fig. 4, chronically inflamed pericyst infiltrated with eosinophil-dominant, mixed chronic inflammatory exudates. Fig. 5, multiple protoscolices.

**Figs. 7 and 8:**
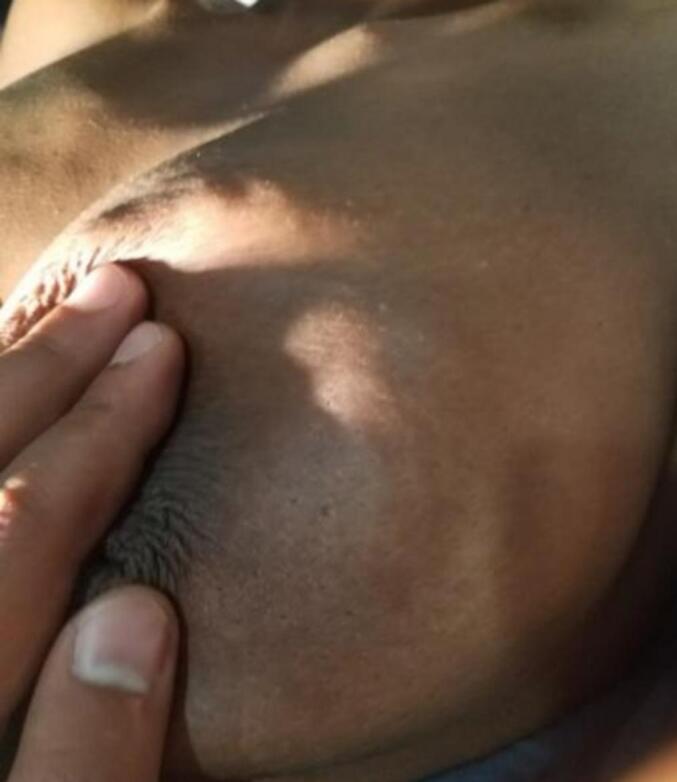

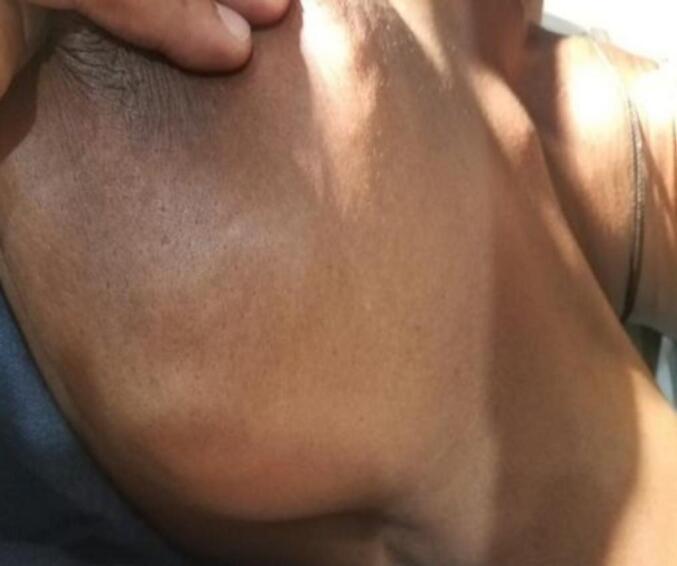
show the breast of a patient with a hydatid cyst that mimics breast cancer, exhibiting a peau d'orange appearance, hard consistency, and fixation to the skin.

**Figs. 9 and 10:**
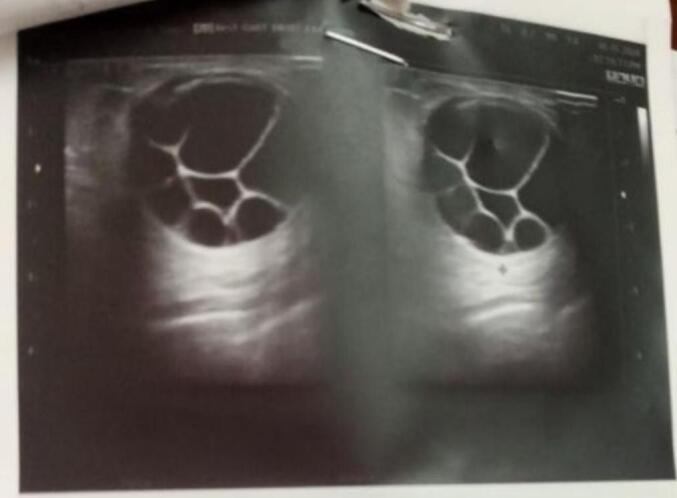

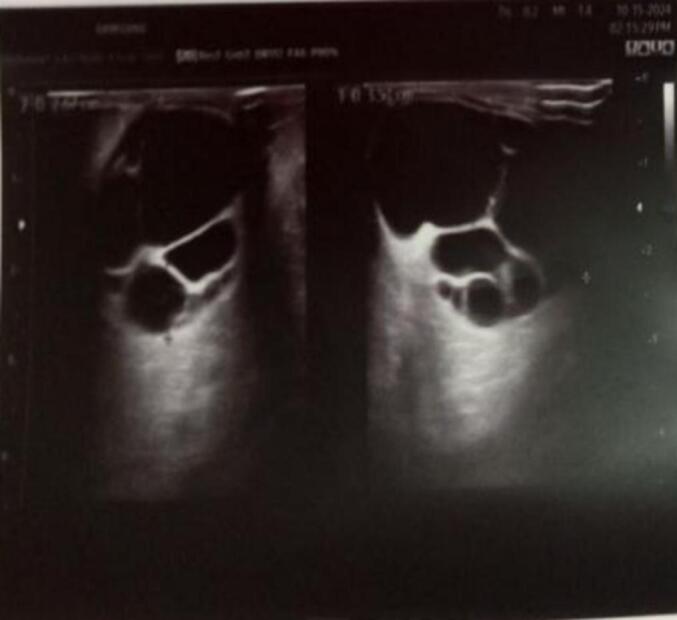
show a 3 by 4 cm anechoic mass with double-layered, doughy cystic breast lesions secondary to hydatic cyst.

## Guarantor

Dr. Misganaw Ayenew Amogne

## Ethical approval and consent to participate

This research study has been designed in accordance with ethical guidelines to ensure the protection of participants' rights and welfare. Prior to the commencement of the study, ethical approval was obtained from the Ethics Committee of Debre Markos University. All potential participants will be provided with an Informed Consent Form that outlines the purpose of the research, procedures involved, potential risks and benefits, and measures taken to ensure confidentiality.

## Consent for publication

Written informed consent was obtained from the patient for publication of this case report and any accompanying images. A copy of the written consent is available for review by the Editor-in-Chief of this journal.

## Declaration of Generative AI and AI-assisted technologies in the writing process

During the preparation of this work the author(s) used [CHATGPT] in order to improve language and readability. After using this tool, the author(s) reviewed and edited the content as needed and take full responsibility for the content of the publication.

## Funding

This case report received no external funding.

## Author contribution

Dr. Misganaw Ayenew Amogne, a corresponding author.

Other authors

1. Dr. Muluken Getinet Mekuriaw was a member of the patient management team and contributed to the review and editing of the case report.
2. Dr. Dagimawi Abiy Abate was a member of the patient management team and contributed to the review and editing of the case report.
3. Dr. Yabibal Tsegaye Workineh was involved in direct patient care, follow-up, and documentation of case reports as well as writing.
4. Mandante Bogale Tiruneh was a member of the patient management team and contributed to the review and editing of the case report.

## Conflict of interest statement

The authors declare that they have no competing interests related to this study. This includes any financial, personal, or professional relationships that could be perceived to influence the outcomes or interpretations presented in this manuscript.

## Data Availability

The data that support the findings of this study are available from the corresponding author upon reasonable request. Due to privacy and ethical considerations, access to the raw data will be granted only after a formal request is made, and appropriate measures will be taken to ensure that participant confidentiality is maintained.
